# Elder Abuse from Life Course Perspective: Effects of Childhood Victimization and Mediating Role of Social Factors

**DOI:** 10.1093/geroni/igaf122.3139

**Published:** 2025-12-31

**Authors:** Louis To, Elsie Yan, Xue Bai

**Affiliations:** The Hong Kong Polytechnic University, Hong Kong, China (People’s Republic); The Hong Kong Polytechnic University, Hong Kong, China (People’s Republic); The Hong Kong Polytechnic University, Hong Kong, China (People’s Republic)

## Abstract

Developing effective intervention strategies for elder abuse requires a comprehensive understanding of its risk and protective factors. Since aging is a continuous process, elder abuse should be understood from a life course perspective. The present study aims to explore the mediating effects of ageism, family functioning, and satisfaction towards social policies and environments on the relationship between childhood victimization and present elder abuse experience, using a life course approach. A total of 5,007 persons aged 60 or above participated in this cross-sectional quantitative survey in Hong Kong. A significant and positive total effect (𝛽=0.199, SE = 0.007, t = 28.726, CI [0.186, 0.213]) was found, indicating that experiences of childhood victimization were associated with more frequent elder abuse. The mediation analysis identified significant and positive indirect effects of child victimization through family functioning (𝛽=0.010, SE = 0.002, CI [0.006, 0.015]), ageism (𝛽=0.056, SE = 0.005, CI [0.047, 0.065]), and social and policy environments (𝛽=0.004, SE = 0.002, CI [0.001, 0.008]) on current elder abuse experiences. Childhood victimization is associated with more frequent elder abuse experiences among older adults who experienced high levels of ageism, family dysfunction, and dissatisfaction with social and policy environments. The significant direct effect (𝛽=0.129, SE = 0.007, t = 17.719, CI [0.115, 0.143]) indicates that these factors serve as partial mediators. Our results confirmed the lifelong detrimental impacts of childhood victimization. Addressing ageism, family function, social and policy environment may provide a possible point of intervention to mitigate the long-term impact of childhood victimization and prevent elder abuse.

